# Human Leukocyte Antigen (HLA) and Immune Regulation: How Do Classical and Non-Classical HLA Alleles Modulate Immune Response to Human Immunodeficiency Virus and Hepatitis C Virus Infections?

**DOI:** 10.3389/fimmu.2017.00832

**Published:** 2017-07-18

**Authors:** Nicole B. Crux, Shokrollah Elahi

**Affiliations:** ^1^Faculty of Medicine and Dentistry, Department of Dentistry, University of Alberta, Edmonton, AB, Canada; ^2^Faculty of Medicine and Dentistry, Department of Medical Microbiology and Immunology, University of Alberta, Edmonton, AB, Canada

**Keywords:** major histocompatibility complex, human leukocyte antigen, classical human leukocyte antigens, non-classical human leukocyte antigens, human immunodeficiency virus, hepatitis C virus and immune regulation

## Abstract

The genetic factors associated with susceptibility or resistance to viral infections are likely to involve a sophisticated array of immune response. These genetic elements may modulate other biological factors that account for significant influence on the gene expression and/or protein function in the host. Among them, the role of the major histocompatibility complex in viral pathogenesis in particular human immunodeficiency virus (HIV) and hepatitis C virus (HCV), is very well documented. We, recently, added a novel insight into the field by identifying the molecular mechanism associated with the protective role of human leukocyte antigen (HLA)-B27/B57 CD8^+^ T cells in the context of HIV-1 infection and why these alleles act as a double-edged sword protecting against viral infections but predisposing the host to autoimmune diseases. The focus of this review will be reexamining the role of classical and non-classical HLA alleles, including class Ia (HLA-A, -B, -C), class Ib (HLA-E, -F, -G, -H), and class II (HLA-DR, -DQ, -DM, and -DP) in immune regulation and viral pathogenesis (e.g., HIV and HCV). To our knowledge, this is the very first review of its kind to comprehensively analyze the role of these molecules in immune regulation associated with chronic viral infections.

## Introduction

**Figure 1:**
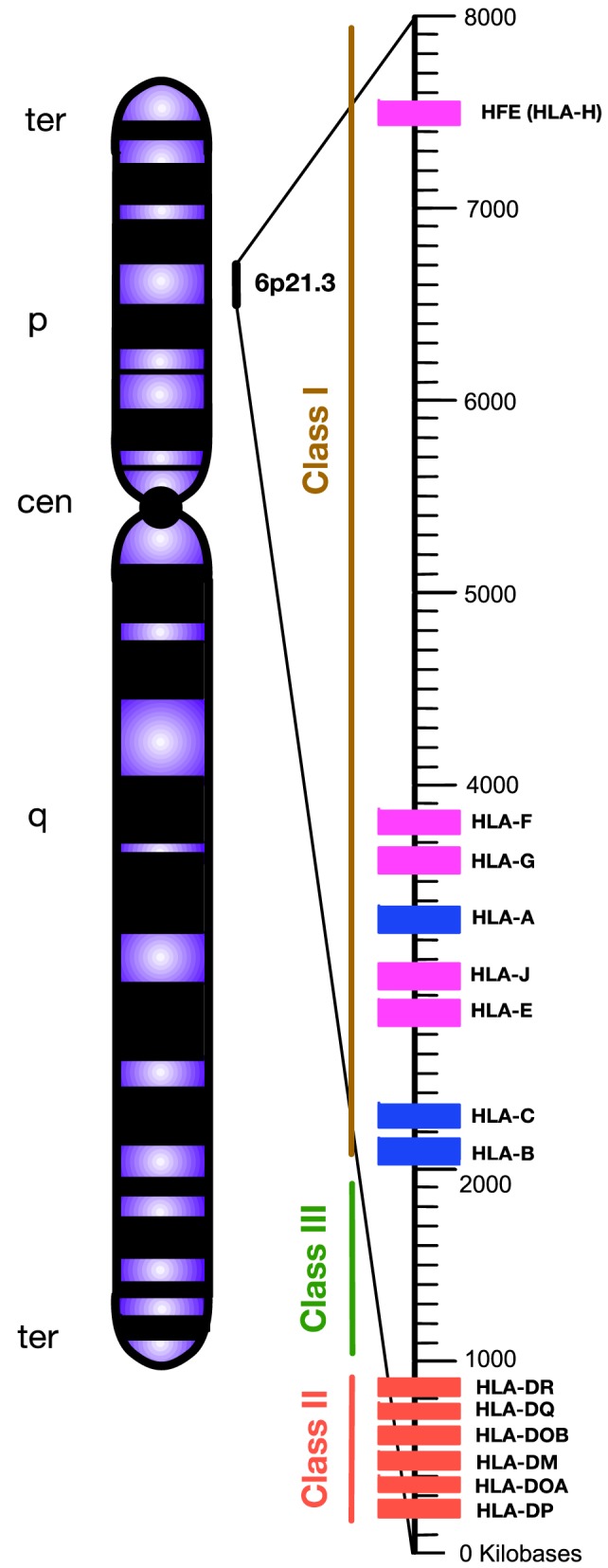
Simplified diagram of the position and organization of human leukocyte antigen (HLA) genes on human chromosome 6. HLA-class I encompasses “classical” HLA-Ia and “non-classical” HLA-Ib loci, which are differentiated by blue and pink, respectively. HLA-class II are encoded by various HLA-II loci labeled by red. Unlike HLA-class I and II, the HLA-class III region does not encode HLAs. Instead, this densely packed region encodes for various inflammatory molecules, complement, and heat shock proteins. Telomere, ter; p-arm, short arm; q-arm, long arm; Centromere, cen.

Chronic viral infection imposes major selective pressure on the host’s immune system. Previously, it has been shown that susceptibility and/or disease progression in a patient with a chronic viral infection, such as hepatitis C virus (HCV) or human immunodeficiency virus (HIV), is influenced by host genetic factors (1, 2). Following their discovery in the 1970s (3), human leukocyte antigen (HLA) loci appeared to be a leading genetic candidate for infectious disease susceptibility. HLAs are classified as the major histocompatibility complexes (MHCs) because of their important role in enabling the immune system to recognize “self” versus “non-self” antigens (4). HLA in human is analogous to the MHC in other animals such as mouse. The classical HLA loci consist of class Ia (HLA-A, -B, -C), class Ib (HLA-E, -F, -G, -H), and class II (HLA-DR, -DQ, -DM, and -DP), which are involved in antigen presentation to CD8^+^ T cells, natural killer cells (NK cells), and CD4^+^ T cells, respectively (5, 6). They are encoded in a ~3,500 kb segment on human chromosome 6p21.3, which is the most variable region in the human genome (7) (Figure 1).

**Figure 2:**
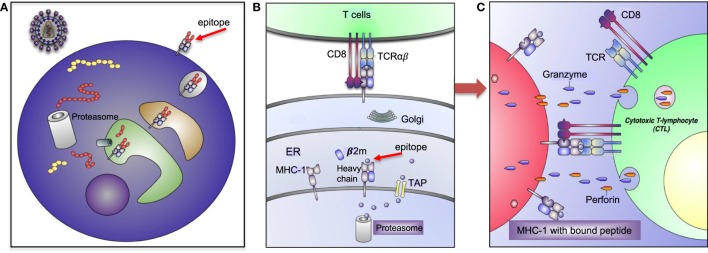
Human leukocyte antigen (HLA)-I antigen-processing and presentation pathway. **(A**) This model depicting how a pathogen (e.g., human immunodeficiency virus) is taken up and processed into small peptides (8–16aa) before binding to HLA-I molecule. **(B)** HLA-I is assembled in endoplasmic reticulum (ER) and consists of heavy chain and β-2 microglobulin (β2M). Proteins and antigen are degraded/processed by cytosolic and nuclear proteasomes resulting in short peptides called epitopes. Epitopes are translocated into the ER *via* transporter associated with antigen presentation (TAP). These peptides may require additional trimming in the ER prior to binding to HLA-I molecules. Eventually, epitope:HLA complex will be transported *via* the Golgi and presented on the cell surface of CD8^+^ T cell. **(C)** Recognition of the epitope:HLA-I complex *via* T cell receptor (TCR) activates cytotoxic T cell (CTL) that releases perforin and granzyme B (GzmB) to eliminate the infected cell.

Human leukocyte antigens are one of the most polymorphic genes in humans, with several thousand alleles encoding for functional polypeptides (8, 9). This high level of polymorphism and heterozygosity enable the immune system with a selective advantage against the diversity of microorganisms and antigens the host encounters. However, extreme polymorphism and possible mutations in the MHC increases the chance of autoimmune diseases (10). Thus, these molecules play an important role in the regulation of host’s immune response as presenters of self-and/or foreign peptides/antigens to T cell receptors (TCRs) for initiating of tolerance and cytotoxic T cell (CTL) or helper T cell response (11) (Figure 2).

Human NK cells mainly use a group of germ-line encoded killer cell immunoglobulin-like receptors (KIRs) to interact with HLA-class I molecules as their major ligands (12). Expression of HLA-I-binding inhibitory receptors (e.g., KIR, NKG2A/CD94, ILT2, and LIR1) results in tolerance of NK cells toward normal cells. The NK cells usually attack abnormal cells that show down regulation of surface HLA-I molecules, termed “missing self recognition” (13). However, “missing self” results in susceptibility to licensed NK cells, which lyse cells lacking HLA-I (14). In rare cases, such as certain tumor cells and viral infected cells, loss of HLA-I/MHC-I expression occurs as a mechanism of avoiding CD8^+^ T cells response; in these circumstances, NK cells can eliminate HLA-I/MHC-I-deficient cells (15). Therefore, HLAs present host peptides allowing for “self” recognition and evasion of autoimmunity, as well as licensing of NK cells (8). Classical HLA molecules have unique functions in cell–cell interaction and immune response, pending on their expression levels, physical structure, and peptide affinity (16). This has led to the identification of crucial role for HLA alleles in adaptive immune response alongside the innate immune response in viral control (17). Thus, certain combinations of KIR and HLA genes may contribute into the susceptibility of host to viral infections, autoimmune disorders, and even cancers.

Currently, a large body of evidence indicate a genetic association between classical HLA loci and important infectious diseases, such as HIV, hepatitis, malaria, tuberculosis, leprosy, leishmaniasis, and schistosomiasis (18–21). Although there are some inconsistencies in the interpretation of data for bacterial and parasitic infections, the role of HLA alleles in HIV infection and HCV has been widely documented. In the context of viral infections, HLAs have been associated with events, such as drug hypersensitivity, spontaneous clearance, or viral persistence in HCV, as well as better disease outcome in some HIV-infected individuals defined as elite controllers (ECs) and/or long-term non-progressors (LTNPs) (22–24). Different groups have defined LTNPs in different ways but, in our studies, we have defined them as individuals who have been infected with HIV more than 10 years, showing low detectable plasma viremia (<10,000 HIV-RNA copies/ml), CD4 count of ≥500, and remain antiviral therapy naïve (22). ECs or natural controllers have normal CD4 count, plasma viral load persistently below 50 copies/ml, and treatment naïve (25).

Specific HLA alleles and their cognate epitopes have been associated with viral clearance or susceptibility in individuals based on preadaptation in the host and escape mechanisms from HLA recognition (26). For instance, preexisting adaptation of the incoming virus to certain HLA could influence the disease outcome in patients expressing the appropriate HLA-type. In agreement, the preadaptation of the source sequence to HL A-B*08 has been associated with the reduced ability of the hosts with HLA-B*08 to control the virus (26).

Recently, we described how CTLs restricted by HLA-B*57 and HLA-B*27 alleles are responsible for superior control of HIV as being independent of epitope specificity of CD8^+^ T cells (22). These collective data suggest a model of natural immunity and/or immune robustness in virus-infected individuals with specific HLA-types, or conversely, certain genotypes may result in susceptibility to disease progression. For instance, HLA-B*35Px has been shown to be associated with rapid disease progression in HIV-infected individuals (27). Therefore, HLAs play a complex role in immunomodulation during chronic viral infections and interact in epitope-dependent or independent pathways to mediate immune responses. In addition to viral infections, the role of HLAs in conditions, such as autoimmune diseases, malignancies, and allogeneic responses, has also been widely investigated (28–30).

This review focuses on identifying the contribution of classical and non-classical HLA allele group in immunoregulation and appreciating the diverse impact of HLAs in HIV and HCV pathogenesis.

## Specific HLA-A Allele Group Employ Diverse Mechanisms to Either Protect Against Viral Infection or Enhance Viral Persistence

During chronic viral infections, three qualities of CTLs lead to a successful antiviral response. First, if the CTL targets a conserved as opposed to a variable viral region/epitope, it will intuitively be superior in viral control (22, 31–33). Another ability that enhances the efficiency of viral clearance is polyfunctionality. For example, in LTNPs CTLs are more polyfunctional and, thus, more effective in HIV control (22, 32–34). Third the proliferative capacity, presumably proliferation of CTLs, links to their ability to kill infected targets. This is also well exemplified in LTNPs and ECs (32, 35). By contrast, targeting variable regions/epitopes, lack of polyfunctionality, and/or poor proliferative capacity can lead to an unfavorable outcome (31). Therefore, if a HLA allele is able to influence any of these characteristics, this may lead to either a protective or unfavorable immune response.

The role of HLA-A allele group in HIV-1 infection is less appreciated than HLA-B alleles, possibly due to observed differences in their CTLs function (32, 34, 35). The HIV-1 protein Nef downregulates HLA-I and, thus, prevents HLA-I molecule from presenting antigens to the cell surface and evades host immune surveillance (36). However, it appears that HLA-A cytoplasmic domains are more susceptible to HIV-1 Nef-mediated downregulation than HLA-B cytoplasmic domains (37). Thus, relative resistance to Nef-mediated downregulation by the cytoplasmic domains of HLA-B might be a contributing factor for better disease outcome in HLA-B^+^ individuals. This raises the question of whether higher polyfunctionality, more avidity and greater clonal expansion of HLA-B restricted alleles could be in part due to HIV Nef evasion in these individuals or other factors enabling HLA-B restricted CTLs confer better protection against HIV-1 infection. Although the role of HLA-A alleles in disease protection is debatable, some studies indicate certain HLA-A variants, such as HLA-A*03 and HLA-A*30, are associated with effective antiviral therapy. For instance, HLA-A*03 is associated with spontaneous clearance of HCV and HLA-A*25 with unfavorable treatment outcome in HCV infection (17, 38). In the context of HIV infection, HLA-A*03 has been shown to be dominant in targeting CTL epitopes in both Nef and p24 regions, but HLA-A*02 favors the amino acids in the p24 dense region (39). Therefore, this highlights the role of HLA-A types in selecting the hydrophobic and conserved amino acid positions within Nef and Gag proteins.

**Figure 3:**
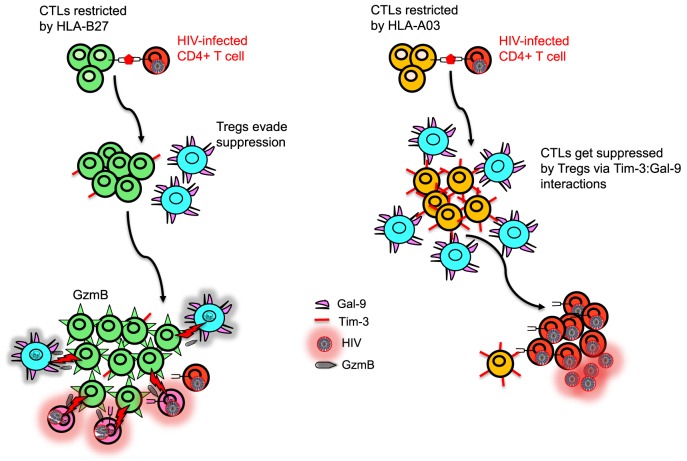
Model depicting differential regulation by CD8^+^ T cells restricted with different human leukocyte antigen (HLA) alleles. HLA-B27 and/or B57-restricted CD8^+^ T cell do not upregulate T-cell immunoglobulin and mucin domain-containing molecule 3 (Tim-3) upon recognition of their cognate epitopes on human immunodeficiency virus (HIV)-infected CD4^+^ T cells, whereas HIV-specific non-B27/B57 such as A03-restricted CD8^+^ T cell upregulate high levels of Tim-3. Regulatory T cells (Tregs) express Galectin 9 (Gal-9) and suppress and kill A03-restricted CD8^+^ T cells due to their high expression of Tim-3 but cannot suppress proliferation of B27-restricted CD8^+^ T cells. Polyfunctional B27-restricted CD8^+^ T cells upregulate high levels of granzyme B (GzmB) and kill not only infected CD4^+^ T cells but also Tregs that they encounter. Therefore, B27-restricted CD8^+^ T cells are capable of keeping HIV replication in control during chronic infection, whereas A03-restricted CD8^+^ T cells lack this ability.

Other HLA-A subtypes have also been associated with disease outcome. For instance, among HIV seroconverters in Zambia, HLA-A*36:01 has been associated with high viral load. By contrast, HLA*74:01 has been associated with low viral load (40). Similarly, there has been association of HLA*74:01 with lower viral load, higher CD4^+^ count, and overall strong protection from HIV-1 acquisition and disease progression in a cohort in Tanzania (41). Although the mechanism of protection is unknown, there is a possibility that HLA-A*74:01 might be in strong linkage equilibrium with another protective polymorphic gene in this population. Alternatively, HLA-A*74:01 may prime the innate immune response against HIV-1 infection. Because HLA-A*74:01 shares common peptide-binding properties with HLA-A*32:01 which has been associated with lower viral load in Caucasians (42). HLA-A*32:01 serves as a ligand for KIR3DL1 and KIR3DS1 on NK cells and, thus, may act prior to the onset of the adaptive immune responses without need for priming (41). A functional supertype, including HLA-A*02:02, HLA-A*02:05, HLA-A*02:14, and HLA-A*68:02, has been reported to be strongly associated with low risk of HIV-1 transmission in female sex workers, suggesting that different mechanisms are involved in disease acquisition versus progression (41, 43). Despite polymorphic nature of HLA molecules and different classifications, HLA-I molecules can be clustered into groups, defined as supertypes which reflects some common structural features of the peptide-binding grooves of these HLA-I molecules (44). By contrast HLA*23:01 has been associated with increased risk of HIV-1 transmission, but HLA*24:02 marginally was correlated with decreased risk of HIV-1 seroconversion (43). In addition, HLA-A*23:01 has been reported to be associated with accelerated disease progression to AIDS in a cohort of white homosexual men (45) and young children (46). Although the underlying mechanism for these observations are unknown, one can reasonably argue that protective alleles may present highly conserved HIV-1 epitopes to elicit stronger protective cellular immunity. In agreement, women in this study exhibited vigorous CTL responses to highly conserved epitopes presented by HLA-A*02:02 and HLA-A*68:02 (43). Alternatively, HLA alleles that tolerate more epitope variation could be associated with protection and those that are sensitive to mutation could be linked to increased risk of disease progression (47). In our studies with a wealth of functional data, no protective role for CTLs restricted by a variety of HLA-A alleles (e.g., HLA-A*03, A*24, A*25, A*02) was implicated (22). More importantly, we discovered that CTLs restricted by HLA-A alleles, in particular HLA-A*03, express significantly higher levels of T-cell immunoglobulin and mucin domain-containing molecule 3 (Tim-3) compared with CTLs restricted by HLA-B alleles and become suppressed by regulatory T cells (Tregs) and, thus, are associated with bad disease outcome in the context of HIV-1 infection (22) (Figure 3). In addition, we found that CTLs restricted by HLA-*A02 group recognizing herpes simplex virus (HSV-2) or Epstein–Barr virus (EBV) epitopes were also susceptible to Tregs-mediated suppression (22). This indicates that suppression of CTL proliferation by Tregs is dependent on the HLA allele restriction, but independent of the epitope specificity of the suppressed cell. With regard to HCV infection, HLA-A*01 and HLA-A*02 have been shown to confer unfavorable outcomes over the course of infection with a poor response to antiviral therapy, HLA-A*24 displays a somewhat favorable response, and HLA-A*25 is indicative of a poor IFN treatment outcome (38). However, no HLA associations to our knowledge have been reported with current direct acting antiviral HCV drugs. Another associated protective factor is viral polymorphism in NS3 and NS5B proteins of HCV as reported in an Australia cohort (26). This suggests that viral epitopes in these regions are important in disease control and escape within these target epitopes could impact disease outcome. Similarly, HLA-A*09 plays a protective role against HCV and correlates with low viral load, whereas HLA-A*11 has been reported to be associated with viral persistence (48).

## How Do HLA-B Supertypes Confer Protection Against HIV/HCV Infections?

A significant body of functional and genetic evidence has implicated CTL response as the major determinant of disease outcome following presentation of HIV-1 peptides by HLA-class I molecules (49). These immunogenetic effects have been found through large association studies, with the HLA-B alleles; HLA-B*08, HLA-B*35, HLA-B*53, HLA-B*55, and HLA-B*56 being correlated with worse disease outcome (27, 50, 51), while HLA-B*27 and HLA-B*57 are enriched in HIV controllers (32, 52, 53). The first HIV genome-wide association study (GWAS) demonstrated a major role for HLA-B*57 in maintenance of CD4^+^ T cells and reduction of viral load (54). Overwhelming evidence in support of this has been reported by different groups (22, 32, 45, 53). Although the protective role of HLA-B*57 has been observed mainly in HLA-targeted studies and in GWAS, it should be noted that the majority of HIV-1-infected patients having HLA-B*57 eventually progress to disease at a rate slower to that of those without HLA-B*57 (55). Although the mechanism(s) of disease progression in these individuals is not fully understood but our longitudinal analyses in HLA-B*57 allele group confirmed that when clinical progression to disease occurs, HLA-B57-restricted HIV-specific CTLs lose their ability to evade Tregs (22). However, it is impossible with these observations to determine whether disease progression in B57^+^ individuals is a cause or effect of loss of ability to evade Tregs. No single genetic variant yet identified uniformly confers elite control of HIV-1, and it is likely that additive or synergistic effects of multiple genetic variants are required for HIV-1 control, as seems to be the case for HLA-B*57. In agreement, another GWAS implied that diversity at the HLA-B gene locus is the primary host genetic influence and identified key amino acid positions in the peptide-binding groove of HLA-B which were strongly associated with HIV-1 control (56, 57). Furthermore, it has been reported that the magnitude of the cross-reactive CTL repertoire negatively correlates with viral load as individuals with a more cross-reactive CTL repertoire control viral loads better during the acute phase of the infection (57). Thus, enhanced cross-reactivity of the T cell repertoire restricted by HLA-B*57 that binds to a few self-peptides leads to greater immune pressure on the infecting and emergent HIV-1 mutants compared to those with T cells restricted by HLA molecules that bind more types of self-peptides (57). This should also confer protection against other fast-mutating viruses such as HCV (58). By contrast, HLA alleles that have greater binding diversity to self-peptides (e.g., HLA-B*08) or in other words have limited cross-reactive response to immunodominant epitopes are associated with faster disease progression in HIV (59) and HCV (60). Therefore, the correlation between the diversity of peptides presented in the thymus during T cell development may impact disease control or progression. In agreement, T cells restricted by HLA-B*57 that bind to few self-peptides are subject to less rigorous negative selection in the thymus and should be more susceptible to recognizing self-peptides (57). In fact, HLA-B*57 has been associated with autoimmune psoriasis and hypersensitivity reactions (61, 62). Similarly, enhanced cross-reactivity of HLA-B27-restricted CTLs and their unique features (e.g., misfolding, homodimers) may contribute to the enhanced risk of autoimmunity associated [e.g., ankylosing spondylitis (AS)] with this allele (63, 64). As the HLA-B*27 allele group is responsible for about 50% of all genetic susceptibility to AS, other genes may also contribute in the pathogenesis of AS (65).

The immune system has evolved to reduce excessive cytotoxicity by upregulation of inhibitory receptors on activated immune cells (66). For instance, Tim-3 is an inhibitory receptor present on a wide range of immune cells (67) and interacts with Galectin 9 (Gal-9) that is constitutively expressed on Tregs to inhibit CTL response (22). We have shown that CTLs restricted by HLA-B27 and HLA-B57 are able to evade Treg-mediated suppression during chronic infection by killing Tregs they encounter in a granzyme B (GzmB)-dependent manner (22). We have shown that most HIV-specific CTLs restricted by non-B27/B57 HLA alleles upregulate Tim-3 when encounter their cognate epitopes and are, therefore, susceptible to Treg-mediated suppression *via* Tim-3:Gal-9 interactions. By contrast, CTLs restricted by HLA-B27 and HLA-B*57 highly upregulate GzmB (33) but do not express significant levels of Tim-3 upon epitope recognition and, therefore, they are not suppressed by Tregs *via* Tim-3:Gal-9 interactions (22) (Figure 3). Interestingly, we have shown that even healthy and HIV negative individuals with HLA-B27/B57^+^ have lower Tregs frequency (22). As such, evasion of Tregs suppression by HIV-specific CTLs restricted by HLA-B*27 or HLA-B*57 may account for delayed disease progression in individuals restricted by these allele groups (22). Furthermore, we found that resistance to Treg-mediated suppression of HLA-B27 and B57-restricted CTL is irrespective of the epitopes recognized by the T cells, suggesting that resistance to Treg-mediated peripheral tolerance is related to the allele restriction rather than the epitope specificity. In agreement, HSV-2 or EBV B27-restricted CD8^+^ CTL evade Treg-mediated suppression, whereas HSV-2- and EBV-specific CD8^+^ CTL restricted by other alleles were susceptible (22). This phenomenon acts as a double-edged sword, as evading Tregs-mediated suppression could be beneficial for the host in chronic viral infections but may mediate autoimmune conditions (68, 69). The mechanisms responsible for reduced upregulation of Tim-3 and increased levels of GzmB upon recognition of their cognate epitopes by B27 and B57-restricted CTLs compared with other HIV-specific CTLs upon recognition of their cognate epitopes are unknown. Understanding the TCR signaling by CTLs restricted by different HLA alleles will be important to understand the mechanism for potential therapeutic interventions that “switch on” virus-specific CTLs that are restricted by other HLA alleles. Similar studies need to be conducted in cancer patients to determine whether individuals with HLA-B*27 and HLA-B*57 alleles are more protective than the others. Thus, our finding may in part explain why the HLA-B*27 and HLA-B*57 allele groups are also associated with resolution of other chronic infections (e.g., HCV) and why both HLA-B*27 and HLA-B*57 allele groups are associated with autoimmunity.

In addition to being protective in HIV-infected LTNPs (53, 70), these protective alleles are associated with spontaneous clearance of HCV infection (71). Studies have shown that HLA-B*57 provides the greatest protection against HIV progression, and HLA-B*57:01 and HLAB*57:03 are the most common HLA-B*57 allele group in Caucasian and African populations, respectively (72). These HLA alleles seem equally protective and the CTL dominant response in HIV infection is to be the Gag epitope KF11 (KAFSPEVIPMF) (72). In addition, HIV restriction factors, such as AOPBEC3C, APOBECC3D, TRIM26, TRIM32, and CTR9, are elevated in elite controls with these protective HLA alleles (73). Increased expression of antiviral genes, including APOBEC3A, APOBEC3B, ISG15, and BST-2/tetherin are also reported in HLA-B*57 positive healthy individuals (73), suggesting another mechanism of protective immunity against HIV-1 that falls outside of classical HLA-mediated functions.

Despite some similar functionality, HLA-*B57 and HLA-B*27 alleles are completely different. HLA-B*27 has a tendency to misfold that was discovered serendipitously during mutagenesis studies (74) and associated with autoimmune diseases, such as AS, psoriasis, reactive arthritis, and inflammatory bowel disease (75). This allele has three unusual features, distinct from that of a typical molecule of HLA-B allele; altered peptide repertoire, tendency to misfold, and often forms heavy chain homodimers during cell surface recycling (76). More mechanistic studies are required to better understand the intrinsic biological consequences of misfolding, the recognition of the abnormal heavy chains by immune effectors, as well as the peptide presentation of an altered repertoire to CTLs.

As we discussed above, CTLs play a major role in the protective effects of HLA-B*57 and HLA-B*27 in early HIV-1 infection and CTLs restricted by these alleles better define disease progression than HLA-genotype alone (77). However, HIV-1 may escape from CTL responses through selection of mutations that inhibit CTL-mediated killing (78). In HLA-B*57, the major escape of HIV-1 occurs from the immunodominant TW10 (TSTLQEQIGW) epitope through the T242N CTL gene mutation which has a negative effect on viral replication (78, 79). Similarly, the importance of CTLs in patients carrying the HLA-B*27 allele is underscored by the observation that viral escape from CTL epitope targeting KK10 (KRWIILGLNK) is associated with increased viral load and disease progression (80–82). In the context of HCV and HIV, it has been shown that HLA-B*27 presents single immunodominant epitopes, which often results in spontaneous clearance of HCV and slow progression of HIV (45, 60, 83). Recently, it has been shown that the protective HLA-B*27 allele specifically presents CTL epitopes from the NS5B, which harbors the most conserved region of the virus (84). Similarly, HLA-B*27 targets NS5B_2841–2849_ during HCV infections and a conserved p24 Gag epitope in the context of HIV (85, 86). During both infections, escape from the targeted immunodominant epitope is very difficult. Thus, it is thought that the time spent by the virus trying to escape may give the immune response an opportunity to clear it, resulting in a protective phenotype. For HCV escape, two mutations must occur. During the early phase of infection, a single mutation may be selected, but this is not adequate for viral escape. Later in infection, a compensatory mutation outside of the epitope occurs and there is a second mutation at the HLA-B*27 binding anchor of the epitope (82, 87). This epitope is present in the viral RNA-dependent RNA polymerase. The ability to mutate at the main HLA-B*27-binding anchor is dependent on viral fitness (86). Misfolded HLA-B*27 may also be unable to engage KIR3DL1, resulting in increased NK cell activation (88, 89). It has been recently shown that HLA-B*27 only is protective in individuals infected with HCV 1 genotype. This is due to the virus containing the conserved NS5B_2841–2849_ epitope. This explains why HLA-B*27 only appears to be protective in certain individuals, as HCV genotypes other than genotype 1 lack this epitope (90). In addition, HLA-B*27 exhibits a strong “HLA footprint” in a novel HCV NS3 epitope (TVYHGAGTK), which is predominantly targeted in women (23), highlighting sex-specific differences involving the mechanism of viral control and clearance.

As previously mentioned, HLA variants can also cause unfavorable outcomes in chronic infection. A more recent study has suggested that HLA-B*15:02 may play a protective role against HCV but B*15:01 acts as a risk allele. Similar association was reported between the HLA-B*07:01 and HLA-B*07:05 alleles in this Chinese cohort (91). It appears that the differences in the amino acids encoded by these polymorphic alleles may impact the HLA properties at antigen binding sites and this could contribute to the divergent immune response to the virus and subsequently different disease outcomes.

Human leukocyte antigen-B*35 is usually found in haplotypic association with HLA-C*04 and is the most HLA allele associated with rapid HIV disease progression. Two variants of HLA-B*35 have been identified: HLA-B*35-Py (HLA-B*35:01, HLA-B*35:08) and HLA-B*35-Px (HLA-B*35:02, HLA-B*35:03, HLA-B*35:04, HLA-B*53:01). HLA-B*35-Py binds peptides with a proline (P) anchor residue at position 2 of the peptide-binding region and tyrosine (Y) at position 7 (92). HLA-B*35-Px also bind P at position 2, however, they have much more flexibility in peptide binding at position 9. In addition, both can bind non-conventional peptides (Los Alamos database, http://www.hiv.lanl.gov/). Individuals expressing HLA-B*35-Px, but not HLA-B*35-Py subtype, progress rapidly to AIDS (27). The HLA-B*35-Px molecule B*3503 binds with higher affinity to the inhibitory MHC I receptor immunoglobulin-like transcript 4 (ILT4) on dendritic cells (DCs) that does HLA-B*35-Py molecule B*3501 (27). This preferential binding of B*3503 with DCs is associated with impaired DC functionality *in vitro* and *ex vivo* compared with carriers of the B*3501 allele (27). Thus, inhibitory impulses due to preferential interactions of HLA-B*3503 (Px) subtypes with ILT4 could lead to DC dysfunction and, subsequently, accelerated disease progression in these individuals. Interestingly, rapid disease progression associated with HLA-B*3503 (Px) begins during primary HIV-1 and remains noticeable during the course of infection (77). This might be due to lack of CTL response to HIV infection in HLA-B*35-Px compared to those with HLA-B*35-Py (93). Another possible explanation for accelerated HIV disease progression among individuals with B*35-Px is that these alleles do not bind HIV epitopes and fail to mediate a protective immune response in infected individuals (49). The HLA-B*53 allele is included with B*35Px because of its close phylogenic connection with HLA-B35. HLA-B*5301 is more prevalent than HLA-B*35 in African-Americans and associated with rapid disease progression in this population (92, 94). A more recent study has reconfirmed the role of HLA-B35 allele in HIV disease progression and also indicated that individuals with HLA-B*51 allele exhibit rapid CD4 T cells decline and are more prone to opportunistic infections associated with HIV progression (95).

## Abacavir Hypersensitivity and Long-Term Non-Progression in Individuals with HLA-B*57 Serve as a Paradigm for the Epitope-Independent and Epitope-Dependent Model of Immune Regulation

Although pathogens have exerted selective pressure on HLAs to diversify as a mechanism of immune adaptation, this has resulted in the high potential for drug hypersensitivity reactions (96). These reactions seem to be due to HLA-allotype-specific interactions, stimulating T cells to respond to drug-exposed tissues (97). Abacavir is a widely used reverse transcriptase nucleoside analog widely used to treat HIV and HCV infections. In a fraction of HLA-B*57:01 individuals, abacavir hypersensitivity syndrome (AHS) can occur (98, 99), resulting in systemic disease (62). Asp114Asn and Ser116116Tyr polymorphisms in the HLA-B*57:01 binding region, as well as abacavir-responsive T cells, are sufficient to alter peptide recognition, and (100) shifting of peptide binding to the unique C-F binding site and recognition of mostly smaller, aliphatic residues (96, 101). This shift in peptide repertoire results in a diverse naïve and memory T-cell response (98, 102). Fully understanding the mechanism of AHS might allow for alteration of abacavir’s chemical structure so that non-immunogenic analogs may be generated with retained antiviral potency (103). This molecular model of AHS suggests that HLA-peptide recognition is highly epitope-independent and that differences in the peptide-binding site result in an altered peptide repertoire. In turn, this causes downstream effects different from that of regular HLAs.

## Interaction of Diverse Host KIRs with HLA-C Variants is a Determinant of Viral Disease Progression

Human leukocyte antigen-A, HLA-B, and HLA-C are highly polymorphic and are expressed on the surface of nearly all nucleated cells (26, 104). Engagement of HLA-I is critical for CD8^+^ T cell activation, as well as NK cell licensing, but is not necessary for NK cell activation (105).

Human leukocyte antigen-C has homology with HLA-A and HLA-B, with its α-helix being particularly similar to HLA-B, but it differs on several levels: (1) HLA-C has a highly conserved α1 domain containing the KYRV motif of residues 66, 67, 69, and 76, which is only present in HLA-B*46, and a conserved glycine (Gly) at amino acid 45 (106), (2) four conserved regions in the α2 domain, (3) reduced diversity in its binding region, correlated with a restricted set of self-peptides, unlike HLA-A and HLA-B (107, 108), and (4) HLA-C is expressed at only about 10% on the surface of cells, which is 15–35% lower than its counterparts (109). All HLA-C alleles are divided into two groups, HLA-C1 and C2, depending on the amino acid presented at position 80 of the molecule (110). Due to reduced diversity in the peptide-binding region, it seems HLA-C is more specific for NK cell regulation; whereas HLA-A and HLA–B seem to be more specific for CTL regulation (111).

Human leukocyte antigen-B and HLA-C are in direct linkage disequilibrium and, therefore, it has been suggested that HLA-C may not directly play a role in disease progression itself but may only influence HLA-B expression (112). However, other studies have demonstrated that incorporation of HLA-C into HIV-1 envelope enhances viral infectivity and reduces susceptibility to neutralizing antibodies (113, 114). This effect appears to be linked with changes in viral envelope proteins conformation such as GP120 as this association between HLA-C molecules and viral envelope enhances the infectivity of both HIV R5 and X4 isolates (114). A most recent study has mechanistically confirmed the role of HLA-C in enhanced HIV infectivity (115). This study suggests that although HIV-1 infection does not upregulate HLA-C expression, HIV-1 envelope may facilitate dissociation of β2M from HLA-C, leading to higher levels of HLA-C free chain molecules at the cell surface influencing HIV-1 infectivity (115). In agreement, another study has demonstrated an important role for HLA-C in HIV pathogenesis. In this study, higher surface expression levels of HLA-C has been associated with reduced viral load and maintained CD4^+^ T cells count in European Americans and African cohorts (116). In particular, a single nucleotide polymorphism (SNP) rs9264942, which is 35kb upstream from HLA-C and is associated with expression of HLA-C mRNA, suggested that HLA-C contributes to viral load set point in HIV-1 infection (117). However, this may not be the direct effect of HLA-C or SNP-rs9264942 on HIV-1 control but polymorphic miRNA-148a may be responsible for differential HLA-C expression (118). This −35 SNP shows a functional effect (e.g., insertion or deletion) in the 3′ untranslated region (UTR) of HLA-C that directly impacts expression of different HLA-C allotypes through miRNA-148a recognition (119). Thus, disease-associated haplotypes might enforce their action *via* multiple mechanisms such as the characteristics of peptides they interact with and their expression level. Despite a recent study has NK cells play a crucial role in early defense against viruses and malignancies, as they are able to respond rapidly to pathogens without prior sensitization (120, 121). Although CTLs are the main players in viral control, even a strong CTL response fails during chronic HIV infection (e.g., mutation) and impaired CTL response by HCV leads to a persistent infection (122). In HIV-infected patients with a poor CTL response, NK cells play a significant role in viral control (123). NK cells are able to shape the immune response through production of cytokines and chemokines, as well as through direct cell-to-cell interactions with their stimulatory/inhibitory receptors (124–127). NK cells such as T cells need to discriminate self from altered-self. This education, termed “licensing, arming or tuning” results in maturation of NK cell functional repertoire and mediated *via* the engagement of the MHC-I inhibitory receptors (105). NK cell becomes more responsive when licensed and requires a regulatory mechanism, thus preventing excessive cytotoxicity. The “rheostat model” suggests that cytotoxicity of NK cell, which is acquired through licensing, is dependent on the HLA/receptor combinations and the strength of inhibitory signaling always overrides activating signals (128, 129).

It has been widely reported that HIV Nef downregulates HLA-A and HLA-B (130). However, unlike previously thought, HIV-Vpu downregulates HLA-C (131, 132). Primary HIV-1 clones vary in their ability to downregulate HLA-C, possibly due to whether the hosts CTL or NK cell dominate immune pressure. Therefore, in the context of HIV-1 infection NK cells may not be able to maintain licensure. This could result in a high population of unlicensed NK cells, which are normally superior at undergoing antibody-dependent cytotoxicity (ADCC) to kill virally infected cells (133). However, Vpu antagonizes type I IFN-inducible factor tetherin that enhances the presentation of viral antigens on the surface of infected CD4^+^ T cells, thereby disabling NK-cell-mediated killing *via* ADCC (134–136). In support of this, HLA-C on HIV-infected cells diminishes response by NK cells, but this suppressed responsiveness of NK cells depends on host’s HLA-C genotype but independent of viral strain (137). Interestingly, in rhesus macaques infected with SIV lacking functional Nef, viremia was controlled. However, over time SIV become virulent due to mutation and regained Nef function (138). This suggests that Nef is crucial for virulence possibly due to its ability to downregulate HLA-class I.

Epidemiological and functional studies demonstrated that NK cells play a crucial role in HIV control *via* recognition of infected cells by both activating or inhibitory KIRs (139–141). The expression levels of HLA and KIR molecules on NK cells and also their strength of interactions have crucial impact on NK function (141). These KIR genes are located on chromosome 19q13.4 in the leukocyte receptor complex, expressed on NK cells, some T cells, and interact with HLA-class I molecules (142, 143). They have been widely implicated to influence viral disease progression due to their heritability and high polymorphism (144–146), along with their frequent and diverse interactions with HLAs (147, 148). Cross talk between self-specific inhibitory KIRs and their cognate HLA-I ligands is essential for “licensing” as absence of KIRs for self HLA make NK cells “unlicensed” and hyporesponsive (149). These observations indicate that NK cell ability to secrete cytokine and cytolysis is dependent on MHC-I molecule. Thus, KIRs play an important role in HCV and HIV pathogenesis, as different combinations of KIRs have been widely shown to influence different disease outcomes (150, 151). KIRs can be stimulatory or inhibitory and the presented peptide can further modulate the outcome (152). The nomenclature of KIRs is based on the presence of either two or three C-type-immunoglobulin domains (2D or 3D), as well as a long or short tail (L or S). Long-tailed KIRs possess immunoreceptor tyrosine-based inhibitory motifs, which recruit tyrosine phosphates that are critical to inhibitory activity. Conversely, short-tailed KIRs associate with the transmembrane signaling protein DAP12 that causes NK cell activation. The single exception to this short/long-tailed rule is KIR2DL4, which is expressed on CD56^bright^ NK cells and induces cytokine production rather than cytotoxicity (15). Although HLA-C more extensively interacts with KIRs, some subsets of HLA-A and HLA-B present the HLA-Bw4 epitope, which also interacts with certain KIRs, suggesting that NK cells may principally be regulated by HLA-C (153). For example, HLA-C allotypes recognize KIR2DL2 and KIR2DL3 defined by Asn^80^ (HLA-C1); KIR2DL1, and to a lesser degree some KIR2DL2/3 allotypes, recognize HLA-C allotypes defined by Lys^80^ (HLA-C2) (154). Recently, it has been reported that the avidity of the ligand and receptor (the strength of the interaction) as well as receptor density are functionally important during HIV infection (155). In addition, KIR–KIR ligand combinations impact NK cell education and functional responsiveness (156, 157). Most recent studies have demonstrated a differentiating role for HLA-C in ECs versus LTNPs. These studies have shown that KIR2DS receptors and their HLA-C2 ligands escape miRNA-148a regulation in ECs but not LTNPs (158). Therefore, escaping miRNA-148a regulation in ECs supports a dominant role for NK cell-mediated HIV-1 control.

Viremic HIV-infected individuals display reduced expression of NK cell activating receptors (3, 159–161) but increased expression of NK cell inhibitory receptors (159, 162). As blockade of interaction between HLAs (e.g., HLA-C and HLA-E) and their corresponding inhibitory receptors enhances killing of HIV-infected cells with viral strains that reduce MHC-I expression (163). This indicates that the killing ability of NK cells depends on the capacity of HIV isolate to alter MHC-I expression. KIRs or combinations of KIR and HLA-I have different effects on infectious diseases (e.g., HIV and HCV) outcome. For instance, KIR2DL5 facilitates HIV-1 infection in both sexes but KIR2DL2 only in females, whereas KIR2DS1 facilitates the disease course in intravenous drug users only. By contrast, KIR3DS1 is protective in intravenous drug users and KIR2DL3 in all individuals (164). As such, KIR3DS1 and 2DS1 are associated with resistance to HIV infection, while 2DS1, 2DL5, and 2DL2 genes are correlated with enhanced HIV acquisition. Despite the role of KIRs in HIV-1 disease progression, their role in disease acquisition has not been fully supported (165). However, KIR2DS4*001, the only allele of KIR2DS4, has been associated with higher viral load and accelerated HIV-1 transmission in a heterosexual cohort in Zambia (166). Different mechanisms might be associated with differential roles for KIRs in viral infections. For example, KIR2DL2 binds virus-derived peptides on HLA-C resulting in reduced NKC function and a selective advantage for the virus to escape the protective role of this KIR (139, 167). KIR2DL3 binds HLA-C with lower affinity resulting in weaker NK cell inhibition and, subsequently, a protective role for KIR2DL3:HLA-C (168), which explains how subtle differences result in the differing effects of these KIRs (169). KIR2DL2 promotes HLA-I restricted CTL adaptive immunity as expression of KIRs on both NK cells and CTLs have been associated with shaping the adaptive immune system (170). In agreement, KIR2DL2 expressing CTLs survive longer and, therefore, exhibit prolonged immune protection, as KIR2DL2 homozygotes with HLA-B*57 are almost 5 times more likely to clear HCV infection (171). Likewise, CTLs restricted by HLA alleles associated with bad disease outcome survive longer once they carry KIR2DL2 and, therefore, can be detrimental in chronic viral infection (171). A recent study suggests an alternative mechanism for the observed effects of KIR2DL2 in chronic viral infection (172). In HCV infection, KIR2DL2 enhances the detrimental effects of HLA-C*08 and increases the protective effects of HLA-B*54 and HLA-B*57. As the copy number of KIR2DL2 increases, so does the enhancing effects (171). Interestingly, cells with iKIRs have been shown to survive for a longer period in the presence of chronic stimulation (173). Thus, these NK cells with alleles either promoting or protecting against disease progression will survive for a longer time and their effects will be enhanced (172).

Interestingly, in the majority of HIV-infected individuals, there is an increased frequency in HLA-C-specific inhibitory natural killer receptors (iNKRs) in viremic subjects versus aviremic subjects and this upregulation in iNKRs is associated with impaired NK cell cytolytic function (160). In addition, the synergistic effect of KIR3DS1 with HLA-Bw4 to delay HIV progression shows a model of genetic epistasis (174). By contrast, KIR3DS1 alone in the absence of HLA-Bw4 was liked to accelerated HIV disease progression (174). Thus, this lack of protective effects for KIR3DS1 (in the absence of HLA-B Bw4-80Ile) could be associated with other genes in linkage disequilibrium with KIR3DS1. Taken together, the pieces of evidence from different viral infections consistently demonstrate a role for NK cells in viral control. However, viruses can evade NK cell-mediated immune pressure by selecting for variants that prevent the recognition of infected cells by KIRs (e.g., MHC-I alteration).

## HLA-E Signaling Dysfunction as a Viral Strategy to Alter NK Cell Activity and Skewing of Populations

Human leukocyte antigen-E is a non-classical HLA-Ib with broad tissue distribution but least polymorphic of all the MHC-class I molecules. It is upregulated by microenvironmental stresses such as hypoxia and glucose deprivation in tumors (175) but transcribed at lower rates than HLA-Ia molecules (176). Seventeen alleles are known to date with two functional variants, HLA-E*01:01 (HLA-E^107R^) and HLA-E*01:03 (HLA-E^107G^) (177). These variants differ in a single amino acid at position 107 when an arginine (Arg) is replaced by a Gly in HLA-E*0103 (16), resulting in different thermal stabilities and lengths of interaction with cognate receptors (16). HLA-E is mainly occupied by conserved epitopes derived from leader sequences of HLA-class I molecules. The frequencies of HLA-E variants are balanced between populations, though the HLA-E*01:03 variant is usually expressed at higher levels than HLA-E*01:01 (16) This Arg–Gly mismatch may also alter either the conformation of HLA-E or its association with β2M resulting in the presentation of different peptide repertoires (178). In the absence of Tapasin (MHC assembly factor), both variants of HLA-E present drastically different peptide repertories (HLA-E*01:01 presents non-canonical peptides, while under normal physiological conditions and in the absence of Tapasin, HLA-E*01:03 binds non-americ peptides) (179). So, in a transporter associated with antigen presentation/Tapasin impaired environment (e.g., viral infection and tumor), changes in peptide repertoire especially in peptide-binding motif for HLA-E mirrors that of HLA-A*0201(179).

Human leukocyte antigen-E interacts with activating and inhibitory natural killer receptors (NKRs) to regulate NK cell activity. Endothelial cells constitutively express HLA-E and are ubiquitously present along the endothelium of the circulatory system, thus being accessible to NK cells (180). NKRs are made up of CD94, which dictates specificity of NKR interactions with peptides presented on HLA-E, and an isoform of NKG2 that dictates the affinity of the NKR (181). These interactions can either be activating responses (i.e., NKG2C/CD94) or inhibitory responses (i.e., NKG2A/CD94). However, in viral infections, the balance between inhibitory and stimulatory signals could be altered. In murine models, HLA-E also interacts with the TCR (e.g., αβTCR), directly inhibiting CTLs by Qa-1-restricted interaction (182). Thus, HLA-E has a dual role and bridges the innate and adaptive immune response (182).

During HCV infection, natural cytotoxicity receptors, such as NKp46 and NKp30, are downregulated compared to healthy controls (183). The HCV core protein YLLPRRGPRL stabilizes HLA-E expressing cells resulting in impaired NK cell-mediated cytotoxicity. In addition, NKG2A is upregulated on CD8^+^ T cells and NK cells in chronic HCV infection (183). Therefore, enhanced expression of NKG2A on NK cell from HCV patients is associated with decreased cytotoxicity against HLA-E expressing cells and altered NK cell-induced modulation of DC functions in chronic HCV (184). Of note, the two identified HLA-E alleles are HLA-E^R^ and HLA-E^G^, but the HLA-E^R^/HLA-E^R^ homozygous genotype appears to be associated with increased resistance to infection or spontaneous resolution of HCV infection, resulting in an abundance of the permissive HLA-E^G^ in HCV patients (185). Although the exact mechanism of this association remains to be determined, HCV-induces upregulation of the inhibitory receptors NKG2A receptor on both CD8^+^ T cells and NK cell rendering these cells less resistant to inhibition *via* HLA-E/NKG2A interactions as discussed above. Moreover, HLA-E^R^/HLA-E^R^ homozygous genotype is associated with low surface expression of HLA-E, therefore, HLA-E-mediated inhibition might be less efficient in carriers of a homozygous HLA-E^R^ genotype in chronic HCV patients. Taken together, it seems that HCV core amino acids 35–44 will be recognized by CD8^+^ T cells in the context of non-classical HLA-E allele and HLA-E allelic variants impact the course of HCV infection (185). Treatment-induced clearance has also been associated with the HLA-E*01:01 genotype since these patients have a high frequency of antiviral HLA-E-restricted CD8^+^ T cells with increased IFN-γ production, which is correlated with sustained antiviral response in HIV/HCV coinfected patients (186). In this context, Zimbabwean women with the HLA-E^G^ genetic variant (the HLA-E*01:03 allele) are four times less likely to be infected with HIV and interestingly women carrying HLA-E^G^ homozygous and HLA-G*01:05N heterozygous genotypes exhibit 12.5-fold lower risk of HIV infection than those carrying neither genotype (187). It is possible to speculate that individuals restricted by HLA-E^G^ have high levels of the HLA-E molecule with great affinity for viral antigens, which may increase NK cell and CD8^+^ T cell lysis of HIV-infected cells and subsequent protection from infection.

Recently, it was shown that HLA-E presents a highly conserved Gag-derived peptide (AISPRTLNA or AA9) that is unable to interact with NKG2A/CD94, resulting in NK cell-mediated lysis of HIV-infected cells by NKG2A/CD94 NKCs (137). Based on these reports that NKG2A/CD94^+^ NK cells respond more robustly to HIV-infected T-cells than those lacking this inhibitory receptor, which can be speculated that HLA-E on HIV-infected cells is not recognized by NKG2A/CD94. Consistent with this hypothesis, NKG2A/CD94+ NK cells degranulate more extensively than NK cells lacking NKG2A when exposed to K562 cells lacking surface HLA-E (137). Of note, NKG2A/CD94+ CD56^bright^ NK cells, contrary to the popular belief, are more successful at lysing HIV-infected cells than CD56^dim^ NK cells as they possess considerable levels of perforin, granzymes, and granulysin (137). CD56^bright^ NK cells express the high-affinity IL-2 receptor and are, therefore, more active than CD56^dim^ NK cells in the presence of IL-2 (137). CD56^bright^ NKCs commonly express the FcγIIIa receptor and undergo ADCC early during infection. The observation that, overall, CD56^bright^ NK cells are better at lysing HIV-infected cells is consistent with their predominant expression of NKG2A/CD94 and relative lack of KIR expression. By contrast, CD56^dim^ NK cells are less likely to express NKG2A/CD94, but express higher frequencies of KIR2DL receptors and are negatively affected by HLA-C on the HIV-infected cell surface (137). During HIV infection, CD94-NKG2A expressing NK cells decrease dramatically which corresponds with the significant expansion of a distinct population of cells expressing a functional activating CD94–NKG2C receptor and HLA-C-specific inhibitory KIRs (188). This NK cell skewing does not appear to be associated with the viral load but resembles those associated with immune activation and concomitant infections (188). Early in HIV infection, the NKG2A:NKG2C ratio is very low, whereas in the later stages of disease, the ratio becomes much higher and NKG2A gets upregulated in the cytotoxic NK cell subset of HIV-1-infected patients with advanced clinical status (189). Thus, elevated levels of NKG2A might be associated with escape of HIV-infected CD4^+^ T cells from NK cells in late stage of disease since NKG2A-CD94 and HLA-E interactions result in an inhibitory signal. In support, high NK cell diversity results in susceptibility to HIV infection and faster disease progression as repertoire diversity may decrease potency of NK cell-mediated antiviral response (190). Of interest, NKG2C is upregulated in response to HIV infection and it is a major triggering receptor in the response of Vδ1 T-cells to HIV-infected CD4^+^ T cells (191). Early in HIV infection, NKG2C is protective independent of viral strain, as it allows for immediate detection and destruction of HIV *via* ADCC (192). Therefore, upregulation of NKG2C in HIV patients, in combination with genetic-associated NKG2C deletion results in increased susceptibility to HIV infection, higher HIV viral set point, and a faster disease progression, demonstrating a significant role for this activation receptor in HIV infection and progression (192).

## HLA-F Preferentially Interacts with KIR3DS1 to Elicit an Antiviral Response

Human leukocyte antigen-F is the most enigmatic and understudied HLA with tightly regulated tissue distribution (193–195). Tissue expression of HLA-F is controversial, it mainly presents in reticulum endothelium, however, can be upregulated in most lymphocyte subsets upon activation except Tregs (196, 197). Interestingly, other studies were unable to detect HLA-F transcripts in T cell lines but instead reported expression of HLA-F protein only in B cells and tissues containing B cells, such as tonsils and fetal liver (198). It is highly conserved, with 22 reported alleles encoding for four functional variants (199), suggesting a critical role in the immune response depicted by its expression on activated T-cells (200). HLA-F is expressed on monocytes and macrophages, as well on activated CD4^+^ T-cells as an open conformation that does not require β2M or peptide-binding (194, 197, 200). It has been suggested that due to its co-expression with other MHCs, it may have overlapping or interdependent functions with HLA-Ia, or participate in antigen-cross presentation (201).

Recently, HLA-F was found to be a high-affinity ligand for activating NK cell receptor KIR3DS1, leading to degranulation of NK cells and production of antiviral cytokines upon interaction. It has also been liked to delayed disease progression in HIV-1 infection (196). Of note, HIV-1 infection increases the transcription of HLA-F mRNA but decreases its binding to KIR3DS1, indicative of a mechanism for evading recognition by KIR3DS1^+^ NK cells (196). HLA-F also interacts with the leukocyte immunoglobulin-like receptors, such as LILBR1, LILBR2, and lineage II KIRs (195, 202). Although expression of HLA-F is upregulated in healthy activated CD4^+^ T-cells, HLA-F mRNA is also upregulated in HIV-infected CD4^+^ T-cells, but the binding of KIR3DS1 is significantly reduced due to downregulation of HLA-F over time through an unknown mechanism (196). It’s possible to speculate that HIV-1 infection downregulates HLA-F expression on the surface of infected cells but this hasn’t been investigated due to lack of antibody to HLA-F. Interactions of HLA-F with KIR3DS1 inhibit HIV-1 replication *in vitro*; however, HIV control only occurs in HLA-Bw4^+^ individuals. As patients with KIR3DS1, KIR3DL1, and HLA-Bw4 progress slowly to AIDS and have reduced viremia (203, 204). This can be attributed to the synergistic effects of HLA-F:KIR3DS1 and HLA-Bw4:KIR3DL1, the latter of which delays HIV progression (140). KIR3DL1 plays a role in sustained virologic response (SVR) in HCV and HCV/HIV coinfected patients (205), suggesting that during chronic viral infection, a stronger HLA-F:KIR3DS1 interaction is beneficial. Therefore, any host polymorphisms resulting in higher HLA-F or KIR3DS1 expression or increased interaction between two molecules would be beneficial in viral control.

## The Complex Role of HLA-G in Immunomodulation

**Figure 4:**
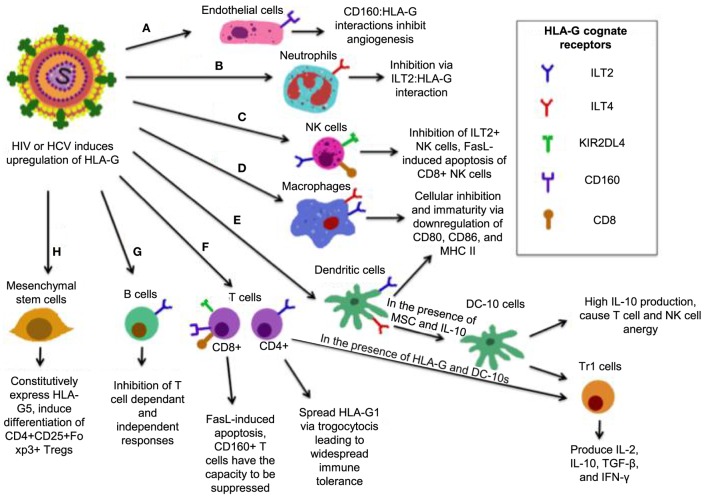
Simplified model of how human leukocyte antigen (HLA)-G mediates its inhibitory functions. **(A)** Interactions between CD160 on endothelial cells with HLA-G inhibit angiogenesis. **(B)** ILT2 expression on neutrophils renders them susceptible to inhibition by HLA-G, resulting in a tolerogenic phenotype. **(C)** The interaction between HLA-G and CD8 on some natural killer (NK) cells triggers FasL-mediated apoptosis, while ILT2:HLA-G impairs NK cell function. **(D)** ILT2 and immunoglobulin-like transcript 4 (ILT4) present on macrophages interacts with HLA-G, resulting a tolerogenic, immature phenotype with reduced activation markers CD80/86, and major histocompatibility complex (MHC) II. **(E)** In the presence of HLA-G, mesenchymal stem cells (MSC), and HLA-G, dendritic cells (DCs) that express ILT2 and ILT4 differentiate into DC-10s, which produce high IL-10 and mediate differentiation of Tr1 cells and immaturity of macrophages, and induce widespread immune tolerance. **(F)** HLA-G:CD8 triggers FasL-mediated apoptosis. KIR2DL4:HLA-G inhibits cytotoxicity, while CD160:HLA-G results in inhibition of proliferation. Upon induction of HLA-G1 on ILT4+ CD4+ T-cells, DC-10 are able to cause their differentiation into Tr1 regulatory cells, which secrete high IL-10 and TGF-β and kill myeloid cells in a Grzm-dependent manner. **(G)** B-cells express ILT2, it is likely that B-cells are susceptible to HLA-G-mediated suppression. **(H)** MSC constitutively express HLA-G5 and *via* HLA-G5 suppress other cells. MSCs also induce CD4+ CD25+ Foxp3+ regulatory T cells (Tregs) *via* cell-to-cell interaction.

Human leukocyte antigen-G belongs to the HLA-class I heavy chain paralogs. It was first characterized by its expression at the fetal–maternal interface, where it was later reported to shield the fetus from the maternal immune system (206, 207). It is distinct from other HLAs in that (1) its coding region is less polymorphic (208); (2) it can be expressed in either an insoluble membrane-bound form (HLA-G1, -G2, -G3, -G4) or a soluble form (HLA-G5, -G6, -G7) (209, 210); (3) it has a highly restricted tissue distribution (211); and (4) it exerts immunosuppressive properties on different immune cells (Figure 4) (212). Soluble HLA-G1 (sHLA-G1) is produced by the proteolytic cleavage of HLA-G1 and is dependent on metalloproteinase activity (213), which is regulated by nitric oxide and the TNF-α/NF-κB pathway (214). sHLA-G1, HLA-G1, and HLA-G5 are the most common forms of HLA-G expressed in healthy tissues (210). HLA-G expression is involved in tumor escape from immunesurveillance (215), tolerogenic responses in transplantation (216), and importantly, blood HLA-G expression increases in viral infections (217–223).

The immunomodulatory activities of HLA-G antigens occur *via* direct interaction with adaptive and other immune cell inhibitory receptors: ILT2/CD85j (expressed on lymphoid cells) (224), ILT4/CD85d (expressed on monocytes, macrophages, and DCs) (225), KIR2DL4 (CD158d) (expressed on NKCs) (226), and CD160 (expressed on endothelial cells, NK cells, and T cells) (227). Though ILT2 and ILT4 may bind other ligands, HLA-G seems to be their receptor of preference than classical HLA-I molecules (228). Therefore, HLA-G through these receptors directly interacts with antigen-presenting cells (APCs), NK cells, and small populations of T cells (229, 230), and exerts its immunoregulatory functions. Of note, HLA-G may utilize its function indirectly by upregulating the expression of HLA-E, which *via* the inhibitory receptor CD94/NKG2A to inhibit both NK cell and T cell functions (231). Although HLA-G can modulate cytokine production by human peripheral mononuclear cells toward a Th2 phenotype, its expression could be upregulated by anti-inflammatory cytokines, such as IL-4, IL-5, and pro-inflammatory cytokines, such as IFN-γ, TNF-α, IL-1β and IL-6 (232), IL-10 (233), and growth factors TGF-β, GM-CSF, and G-CSF (221, 234). Indolemine 2,3-dioxygense (IDO) is another important molecule that plays a role in HLA-G mediated immunoregulation. It has been shown that upon tryptophan depletion, IDO induces HLA-G expression in DCs, induces Tregs, and inhibits HLA-G surface expression in monocytes (235), suggesting a cross talk between IDO and HLA-G expression in immunoregulation.

Human leukocyte antigen-G expression levels are positively correlated with the acceptance of human allograft, suggesting that HLA-G may regulate the function of both CD4^+^ and CD8^+^ T cells (236). For instance, HLA-G interaction with CD4^+^ T cells causes a cell cycle arrest at G1 phase, resulting in inhibition of CD4^+^ T cell proliferation and limiting the induction of Th1 cytokines (237). HLA-G inhibits CTL proliferation and lysis of virally infected cells by interacting with ILT2 (238, 239). The ILT4:HLA-G interaction inhibits monocyte-derived DC maturation. This leads to downregulation of MHC-II and maturation markers CD80 and CD86, and differentiation of DCs into regulatory IL-10 producing DC-10s that cause T cell and NK cell anergy and generation of Tr1 cells (240, 241). Tr1 cells produce high levels of IL-10 and TGF-β, low IL-2 and IFN-γ, and may kill APC *via* perforin and GzmB-dependent manner (242). HLA-G:CD8 interactions induce TCR-independent FasL upregulation and secretion (243), resulting in apoptosis of CD8^+^ T cells and NK CD8^+^ cells by Fas/sFasL interaction (244). Interestingly, mesenchymal stem cells, which express only very low MHC-I level and do not induce T cell activation are able to induce differentiation of naïve T cells into Tregs in the presence of HLA-G (245). B-cell antibody production is also inhibited by HLA-G (246), which may be due to direct effects on B-cells or indirectly by inhibition of helper T cells function (247). HLA-G:CD160 interactions were recently shown to control angiogenesis, which is consistent with vascularization that occurs during pregnancy (248). Neutrophils also have the capacity to be suppressed by HLA-G as they express ILT4 (249). CD160 is also expressed on the majority of NK cells and inhibits them upon HLA-G interaction (249). Similarly, this occurs on CD8^+^ T cells, which may result in CTL exhaustion (250).

Despite the coding region displaying low allelic polymorphism, the HLA-G 5′ regulatory region and 3′ UTR are highly polymorphic. Seven polymorphisms at the 3′ UTR are reported to play a role in HLA-G expression: a 14 bp insertion/deletion (INS/DEL) rs1704 polymorphism (251), and the SNPs +3003 T/C, +3010 G/C, +3035 C/T, +3142 C/G, +3187 A/G, +3196 C/G (252). The 14 bp INS/DEL polymorphism plays a role in alternative splicing, which removes 92 bp, including the region where the 14 bp region is resulting in a higher yield of stable HLA-G mRNA transcripts *in vitro* (253). However, *in vivo* this allele is associated with decreased HLA-G production, deemed the “14 bp paradox” (254, 255). The presence or absence of this 14 bp region alters the set of miRNAs that bind to this locus, thus repressing translation and negatively influencing HLA-G mRNA turnover (252). The only SNP polymorphism reported to alter miRNA binding is the +3142 C/G (256). In this polymorphic site, the G allele favors the targeting of three miRNAs (miR-148a, miR-148b, and miR-152), resulting in reduced HLA-G mRNA production. These polymorphisms exist in linkage disequilibrium (256, 257). The 14 bp (D/D) genotype has been associated with higher levels of vertical transmission of HCV independent from the other non-immunogenetic parameters (258) and HIV-1 (259). In addition, HLA-G*0105N polymorphisms are associated with augmented risk for HIV infection in heterosexual individuals (260). Therefore, maternal HLA-G alleles and/or SNPs that modulate maternal HLA-G expression at the maternal–fetal interface could influence vertical HCV and HIV-1 transmission. Although the exact mechanism of increased vertical transmission is unknown, alter HLA-G expression may influence the function of NK cells, CD4^+^, and CD8^+^ T cells at the fetal–maternal interface and, ultimately, influence the susceptibility to HIV transmission. In addition, the homozygous HLA-G −14-bp (D/D) genotype has been associated with higher viral load, lower CD4^+^ T cell count, and lower survival rate compared with HLA-G +14-bp (D/D) carriers (261). In patients with sickle cell anemia, HLA-G +3142 C/G increases susceptibility to HCV infection (262). In addition, HLA-G genotypes have been associated with susceptibility to HIV infection. HLA-G*01:05N increases in HIV infection, which may be due to lack of HLA-G1 and HLA-G5 expression resulting in compensation by other HLA-G isoforms, which then may inhibit NK cell activation (263). It has also been shown that the HLA-G*01:01:01 genotype is significantly enriched in HIV-resistant women, while HLA-G*01:04:04 is associated with increased rates of seroconversion (264).

Accumulating evidence suggests that during HCV infection, the inactivation of immune effector cells by HLA-G may lead to inability to clear the virus. As CTLs are crucial for restricting viral replication, yet the magnitude of CTL response in chronic HCV decreases dramatically compared with acute HCV (265–267). The tolerizing functions of HLA-G seem to be playing a significant role in viral clearance in non-responders to IFN-α2α and ribavirin therapy. Patients who are able to clear HCV infection express only low levels of HLA-G, while non-responders express drastically higher levels of HLA-G (268). Many patients with high levels of HLA-G who failed to respond to antiviral treatment also had a −1082 G/G genotype, resulting in overproduction of IL-10, higher levels of HLA-G, and inadequate viral clearance (267). In addition, high levels of HLA-G in HCV and hepatocellular carcinoma are associated with shortened overall survival and increased tumor recurrence rate (267, 269). Therefore, it is likely that HLA-G plays a role in virus-associated malignant cell transformation. In agreement, the levels of HLA-G activation positively correlate with the amount of mast cell activation and liver fibrosis (270). For instance, IFN-α upregulates HLA-G, as well as various chemokines and cytokines that are produced around the fibrotic tissue. It is possible that the interaction of HLA-G with IL-T2 on mast cells creates an autocrine loop, and the immune response against HCV-induces liver fibrosis *via* hepatic stellate cells (270, 271). Significant elevation of sHLA-G (sHLA-G1 and HLA-G5) in chronic HCV patients, given its immunotolerant nature, may facilitate persistence of HCV infection (267).

Differential splicing and translation of HLA-G may also alter viral disease progression by various mechanisms. For instance, HLA-G5 is reported to induce TNF-α, IFN-γ, and IL-10 but downregulates expression and function of CCR2, CXCR3, and CXCR4 in different subsets of T cells, while HLA-G1 promotes Th2 polarization of naïve T cells (272–274). There are some important basic questions in biology of HLA-G that merits further investigation. Among them is better understanding how its expression levels can be modulated in different pathological conditions. This knowledge could assist us to induce HLA-G when immune suppression is needed (e.g., pregnancy, organ transplant, and autoimmunity) or to inhibit its expression when can be potentially harmful (e.g., tumor or chronic viral infections).

## Nef-Mediated Downregulation of HLA-H Expression Contributes to Disruption of Iron Homeostasis

**Figure 5:**
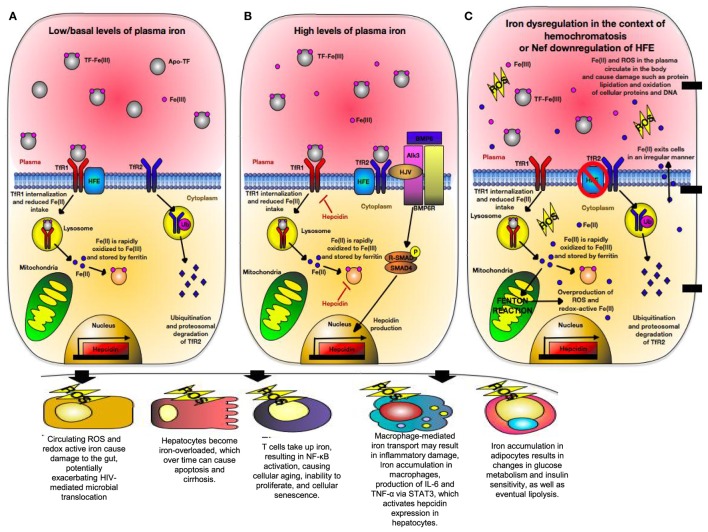
Iron regulation of hepatocytes during normal physiological condition versus conditions of hereditary hemochromatosis (HH) or human immunodeficiency virus (HIV) infection. **(A)** Under normal physiological iron concentration, transferrin-bound iron is taken up upon binding of saturated transferrin to transferrin receptor 1 (TfR1), which is endocytosed and recycled. Fe(III) (indicated in pink) is oxidized to Fe(II) (indicated in blue) in the endosome and is reduced back to Fe(III). Fe(III) then is stored intracellularly by ferritin. TfR1 and transferrin receptor 2 (TfR2) are in competition for *High Fe* (HFE). When not associated with HFE, TfR2 is endocytosed, ubiquitinated, and thus degraded by the proteasome. This maintains uptake of iron into the hepatocyte. **(B)** When too much iron is present in the plasma, HFE interacts with TfR2, preventing its ubiquitination. A multiprotein complex consisting of HFE, GPI-anchor HJV, and TfR2 form on the hepatocyte membrane. HJV is the bone morphogenic protein (BMP) co-receptor, which assists in activating hepcidin gene expression *via* BMP/SMAD signaling. Hepcidin then limits cellular iron intake by promoting degradation of cellular ferritin, as well as downregulating TfR1 expression. **(C)** In situations where HFE is absent (i.e., HIV or certain mutations in HFE), TfR2 is unable to interact with HFE and is thus degraded. In states of high physiological iron, TfR2 is unable to mediate hepcidin gene expression. Due to this, cells become iron overloaded labile cell iron (LCI) and non-transferrin-bound labile plasma iron (LPI). These forms of redox-active iron are able to move across cell membranes in a non-regular manner. LCI participates in a Fenton reaction, resulting in overproduction of reactive oxygen species (ROS) that cause lipid peroxidation, protein nitration, and DNA damage. LPI is able to enter other cells irregularly, thus causing even more production of ROS. Eventually, hepcidin production will be triggered due to STAT3 signaling caused by ROS-induced inflammation.

Human leukocyte antigen-H, also known as the *High Fe* (HFE) gene was discovered in patients with hereditary hemochromatosis (HH) who often have mutated HFE variants (275). HFE is structurally homologous to HLA-A*2 with a wide tissue distribution (276). It is in strong linkage disequilibrium with MHC-Ia, which may be due to competition between HFE and MHC-Ia to bind intracellular components resulting in differential cellular surface expression (277, 278). HFE directly inhibits CD8^+^ T cells *via* their TCR in a peptide-independent manner and interacts with transferrin receptors (TfR) (279). Transferrin receptor 1 (TfR1) and transferrin receptor 2 (TfR2) compete for HFE binding. HFE is expressed strongly with the TfR2 on Kupffer cells, circulating monocytes and macrophages, as well as duodenal epithelial cells (280). At the basal state, HFE:TfR1 interactions inhibit iron overload by lowering the affinity of TfR1 for iron-loaded transferrin and reducing cellular iron uptake (Figure 5A) (280, 281). However, in the presence of high ironbound transferrin, HFE binds TfR2 allowing for cellular iron uptake and hepcidin production (Figure 5B). Hepcidin is a crucial iron-regulatory peptide, which is normally released in the liver in response to iron loading and inflammatory stimuli, such as TNF-α and IL-6 *via* the JAK/STAT3 pathway (282). Once released, hepcidin degrades ferroportin present on TfR1 molecules, preventing further cellular uptake of iron. In monocytes and intestinal cells, HFE inhibits gut iron absorption and macrophage iron efflux *via* a TfR1-independent mechanism, leading to intracellular accumulation of iron, low serum iron, and anemia due to chronic inflammation (283). This occurs *via* TfR2 signaling in which HFE binds TfR2 on the bone morphogenic protein (BMP) receptor type 1 (Alk3) inhibiting its ubiquitination and proteosomal degradation (284). This allows for BMP/SMAD signaling and hepcidin expression (Figure 5B). However, TfR1 signaling occurs through pro-inflammatory-mediated NF-κB signaling (285) and plays an essential role in lymphocyte development (286). Expression of TfR1 is downregulated in iron overload state due to coordination of mRNA-encoding proteins with iron-regulatory proteins and inhibition of TfR1 expression in T cells prevents cell cycle transition from G1 to S phase (287, 288).

One of the first lines of defense against pathogens is the withholding of nutrients deemed “nutritional immunity” with the most significant form being the sequestration of iron (289). Excess iron storage increases the virulence of numerous pathogens such as HIV and inversely associated with survival of HIV-infected individuals (290). During HIV and HCV infections, patients experience iron overload that exceeds the binding capacity of transferrin, resulting in creation of non-transferrable unbound iron (NTBI) that cannot be internalized by TfR1 (291, 292). NTBI is toxic and moves into the cytosol of cells in an irregular manner. It then deposits on the cell surface leading to creation of labile cell iron, which is redox-active and generates reactive oxygen species (ROS) (286). As a result, iron overload alters the balance between ROS production and removal, causing oxidative stress that results in DNA damage, mitochondrial dysfunction, lipid peroxidation, and protein nitration (Figure 5C) (287). Iron also impacts T cells development, for example, iron overload induces a shift in CD4^+^ T cells to a glycolytic metabolism *via* upregulation of the Glut1 transmembrane protein (293). CD4^+^ T cells have a low spare respiratory capacity and, therefore, have insufficient mitochondrial energy to handle stress and viral infection (294). Because energy metabolism is tightly coupled to intracellular redox state, a functional link between glucose metabolism and iron metabolism exists (295). As a result, CD4^+^ T cells exhibit low stress tolerance and HIV intracellular protein Tat101 and Vpr also contribute to additional oxidative stress during HIV infection (296, 297). Therefore, iron level could impact the functionality of CD4^+^ T cells as the prime target for HIV infection.

Unfortunately, the role of HLA-H or HFE has been understudied in the context of viral infections. However, various HFE mutations as seen in HH patients suggest that HFE dysregulation contributes strongly to the particular phenotype seen in viral infections. The most common HFE mutation causing hemochromatosis is C282Y, which results in the inability of β-microglobulin to bind HFE intracellularly and, thus, is not transported to the cell membrane (284). Patients with knockout mutations in HFE have impaired and low CD8^+^ T cell counts (298, 299), modified CD4^+^ T cell polarization (300), and are susceptible to autoimmune conditions such as multiple sclerosis, further showcasing the importance of HLA-H in immune regulation (301).

Like HLA-A and HLA-B; HLA-H contains a conserved motif (^342^YxxA, in which x is any amino acid), allowing its expression to be downregulated by HIV Nef by 90% (302). Of note, Nef protein of HIV-1 plays an important role in HIV pathogenesis. As we discussed in the HLA-A section, Nef is a multifunctional protein and one of its roles is to downregulate surface MHC-I molecules, assisting infected cells to evade CTL response (302). Interactions of HLA-H with HIV-1 regulates cellular iron metabolism, possibly benefiting viral growth (302). In HIV infection, macrophages, microglia, endothelial cells, bone marrow, muscle, and liver are often iron loaded, and degree of iron loading inversely correlates with patients’ survival rate (303). HIV-1 replication exploits the iron-dependent host enzyme ribonuclease reductase (304), which is activated in macrophages *via* altered cellular redox state activating the NF-κB pathway (305). During iron overload oxidative stress highly upregulates NF-κB that acts in synergy with the HIV-1 tat-III gene to enhance viral replication in CD4^+^ T cells (304). Monocytes and macrophages are critical in iron homeostasis and they mediate inflammatory damage to target tissues in lipoatrophy and peripheral neuropathy in iron overload conditions (283, 306). Adipocytes also take up high levels of iron during iron overload (307), altering the expression of various pro-inflammatory cytokines and adipokines, which over time can result in lipolysis (308). This may contribute to the lipoatrophy observed in some HIV-infected individuals and may precede other metabolic complications (309–311). High-fat diets are associated with insulin resistance, modified hepatic lipid and iron metabolism, increased mitochondrial dysfunction, and oxidative stress (312) that are comorbidities associated with both HIV and HCV. Interestingly, the 187C>G HFE SNP in A5005 mice is protective from lipoatrophy (313).

In HIV infection, hepcidin levels are elevated (314, 315). However, hepcidin levels are not associated with severity of anemia, except during coinfection with *Mycobacterium tuberculosis* (316). Degree of surface expression of HFE influences hepatic hepcidin, as well as iron transport (313), consistent with the role of HFE in inflammation (317). In chronic HCV, hepcidin mRNA expression positively correlates with alanine aminotransferase activity and serum iron concentration. Although low expression of hepcidin has no correlation with tissue iron overload in those with chronic HCV, in univariate analysis, HCV viral load and efficacy of antiviral treatment are not significantly associated with hepcidin mRNA expression, suggesting that hepcidin expression is not due to viral load (292).

Macrophages expressing wildtype HFE once infected with HIV accumulated iron, ferritin, and increased cellular HIV-1 Gag expression. However, when this experiment was attempted with Nef deleted HIV-1 or with macrophages from HH patients expressing mutated HFE, iron was unable to accumulate (302). Besides the critical involvement of Nef and HFE in dysregulation of iron homeostasis, individuals with genetic mutation in their HFE gene lack functional HFE molecules on their cells, resulting in impaired iron uptake (Figure 5C).

Human leukocyte antigen-H impacts HCV disease severity and treatment outcome as chronic HCV patients with HFE mutations often have more transferrin saturation and advanced liver fibrosis. For instance, only 27.5% of HFE-mutated carriers achieved response at the end of treatment with pegylated-interferon and ribavirin, as compared to 60% of non-carriers (318), suggesting that individuals with HFE mutations may be more susceptible to chronic HCV due to iron accumulation. In agreement, patients heterozygous for HH infected with HCV displayed higher liver fibrosis than those who were homozygous wildtype, providing additional support that iron dysregulation is a critical component of liver fibrosis (319, 320). However, another study reported no correlation between HFE polymorphisms and HCV in Majorcan patients treated with pegylated-interferon plus ribavirin (321). By contrast, heterozygosity for H63D and/or C282Y HFE gene mutation has been shown to be associated with the lack of SVR to pegylated-IFN and ribavirin combination therapy in patients with chronic HCV (322). Taken together, the MHC-Ib protein HLA-H/HFE is strongly expressed by a variety of cells such as Kupffer cells, circulating monocytes/macrophages and intestinal crypt cells. It plays an essential role in iron homeostasis and C282Y mutation of HLA-H results in protein misfolding, which is associated with the iron overload and as we discussed impacts pathogenesis and treatment outcome of viral infections.

## Diversity of HLA-Class II Genes and Their Influence on Viremia and Viral Dissemination

Major histocompatibility complex-II molecules specifically present antigen to CD4^+^ T cells and the genes encoding them are extremely polymorphic, especially in the peptide-binding groove (323). This renders them highly specific to the epitopes they bind. Genetic correlations between HIV-1 and HLA-II loci have not received as much attention as those observed for HLA-I, indicating more effectiveness of CTL response than humoral immunity in these studies. Although CD4^+^ T cells are preferentially infected by HIV-1, they provide help for the generation of high-affinity antibodies and the function of CD8^+^ T cells and B cells (324), HLA-II genes, specifically functional variants of HLA-DRβ, have been shown to play a fundamental role in the formation of HIV CD4^+^ T cell syncytia (324). However, HIV-infected individuals fail to mount an immune response to this region, possibly due to lack of recognition by the immune system (325). During HIV infection, CTLA-4, CD28, and CD3 are upregulated and assist in syncytia formation with APCs (112). Thus, we may assume that the formation of syncytia with APCs and T cells results in dissemination of HIV from infected to uninfected cells. In addition to CTLA-4, LAG-3 is another inhibitory receptor associated with immune suppression that may function in syncytia to suppress APCs and maintain their immaturity (326, 327). In a study of seroconverted HIV-1 patients, HLA DRB1*13-DQB1*06 was shown to be the only class II haplotype associated with a trend toward increased duration of AIDS-free period (328). Furthermore, inheritance of DRB1*13 alleles has been associated with long-term survival among children with vertically transmitted HIV-1 infection (46). Therefore, the properties of class II molecules, and particularly HLA DRB1*13-DQB1*06, influence HIV-1 pathogenesis and disease outcome. Interestingly, inheritance of the DRB1*13-DQB1*06 haplotype is associated with durable virus suppression and HIV-specific T helper responses (e.g., higher proliferative capacity and IFN-γ production) among early-treated patients (329). Such studies, in which HLA associations are correlated with functional assays, provide strong evidence for the crucial role of certain HLA-II alleles in disease protection. This haplotype may exert its protective effects by presenting crucial epitopes with avid class II binding in highly conserved regions of HIV-1 that may be potentially important targets for HIV-1 vaccines (330). In agreement, patients expressing DRB1*1303 allele exhibit significantly lower mean plasma viral loads and increased CD4^+^ T cell counts, indicating a protective role for this allele in HIV-1 infection (331). The protective role of DRB1*13 has also been shown in other infections, in particular hepatitis B virus infection, where this allele is associated with a higher rate of clearance and/or better clinical disease outcome (332, 333). In addition, studies in human papillomavirus (HPV) infection have associated DRB1*13 with protection against the HPV-16 serotype, and negative correlation of DRB1*13 with the occurrence of cervical carcinoma (334). By contrast, DRB1*13 enrichment has been reported in subjects with autoimmune diseases (335, 336). DRB1*1303 might, therefore, share some similarities with the HLA-Ia allele B*2705, which is associated with protection in HIV and HCV infection but is linked to autoimmune disorders (337). Therefore, it can be speculated that the effect of DRB*13 on disease pathogenesis may be related to particularly effective antigen presentation capacity, which confers protection against viral infections, but may trigger autoimmunity through molecular mimicry with self-antigens (338). It remains uncertain, however, whether the observed associations can be attributed to a stronger and more effective T cell response or whether other mechanisms mediate the DRB1*13 effects. Most recently it has been shown that alleles HLA-DRB1*15:02 and HLA-DRB1*03:01 are significantly associated with HIV control and progression, respectively, and the strength of these HLA-DRB1-mediated effects is independent of HLA-B*57, HLA-B*27, and HLA-B*35Px (330). The protective effects seem to be mediated primarily by the protein specificity of CD4^+^ T cell responses to HIV Gag and Nef proteins (330). The relevance of the genetic background with HCV vertical transmission demonstrate a protective role for HLA-DRB1*04, while HLA-DRB1*10 is reported as a risk factor (339). Interestingly, it was revealed that having a mismatch in the HLA-DRB1 locus between mother and child is a protective factor, indicating that alloreactive immune responses are involved in preventing HCV vertical transmission (339). In addition, HLA-DRB1*04:01 and DRB1*01:01 are shown to be present in patients with spontaneous clearance of HCV, while HLA-DQB1*02:01 is associated with chronic infection (17). In support of these observations, association of other genotypes, such as DRB1*11:01, DQB1*03:01, HLA-DRB1*01:01, and HLA-DQB1*03:01 alleles, with viral clearance in HCV is documented (340). This suggests that MHC-II alleles could present particular viral epitopes to CD4^+^ T cells, resulting in an effective immune response against the virus. However, there have been some contradicting observations regarding the associations of DRB1*01:01and DQB1*03:01 with protection against HCV. For instance, in a study the protective role for DQB1*03:01 was only observed in African-Americans but not in white Americans (341). Interestingly, another study reported a protective role for DRB1*01:01/HLA-DQB1*03:01 against HCV in white Americans but not in African-Americans (342). However, DQB1*07 is associated with viral persistence (343, 344).

## How Does HLA Impact Mother-to-Child HIV Transmission and Progression?

The sharing of HLA alleles between the mother and the offspring occurs routinely in HIV vertical transmission. Mother-to-child transmission occurs *in utero*, intrapartum, or postpartum (breastfeeding) (345). The risk of vertical transmission is associated with the viral load in the mother as higher viral load is the indicative of a non-protective HLA allele (e.g., HLA-B*18, HLA-B*5802, and HLA-B35Px) (27, 346). Thus, HLA alleles may contribute in HIV pathogenesis in early life as defined with rapid CD4^+^ T cells decline, higher plasma viral load, and accelerated disease progression in two different ways (347–349). First, infected children share ≥50% of their mother HLA-type and, therefore, less likely to have protective HLA alleles compared with adults (350). So, this population carries less protective and more non-protective HLA alleles. Second, even if the child possesses a protective allele (e.g., HLA-B*27 and HLA-B*57), this allele cannot protect the child if the vertically transmitted virus has escape mutations within the conserved Gag-epitopes (351, 352). However, a study conducted in a cohort of 61-HIV-infected children in South Africa suggests that HIV-infected children may benefit from protective HLA-B alleles similar to adults, as slow disease progression was associated with the mother or child having one of the protective HLA alleles but more pronounced once they carried non-shared protective HLA-B alleles (346). In addition, they have found that mothers expressing these protective alleles more frequently transmitted escape variants within the Gag-epitopes presented by these HLA-B protective alleles compared with mothers lacking these protective alleles (346). Finally, this study reported that infected children are able to generate Gag-specific CTL response if they carry protective HLA-B alleles without preadaptation of the transmitted virus (346).

Furthermore, the role of HLA-G has also been investigated in a cohort in Zambia and presence of HLA-G 14bp insertion polymorphism has been associated with protection against HIV vertical transmission (259). These pieces of evidence indicate that HLA alleles influence HIV vertical transmission and disease progression in the newborn. Thus, determining the HLA-type of the mother and the child could help to identify children who may experience rapid disease progression.

## Conclusion Remarks

Although the role of HLA alleles in HIV and HCV is well documented. However, some findings have not been confirmed or have been conducted in a very small patient cohort, therefore, these data need to be evaluated with caution. Some differences in the methodology and clinical endpoints might be accountable for some discrepancies reported for some HLA alleles by different groups. To solve these issues, there is an immediate need to conduct similar studies in much larger cohorts especially from different ethnicities, such as African, African-American, Middle-Eastern, and South East Asian populations, to reexamine these observations in these populations. Since the importance role of sex and gender in medical and biologicals studies have become more noticeable, these factors in association with HLA alleles should be taken in consideration. In addition, rigorous functional and mechanistic studies, longitudinal planning, larger sample size and power, reliable statistical analysis, better defining of clinical phenotypes, and reproducibility by different groups are all important elements that will contribute to the confidence of HLA associations with HIV and HCV infection and disease outcome. Another major issue associated with the interpretation of some data is that genes encoding the MHC-I/MHC-II molecules occur within a cluster of other MHC genes with a broad spectrum of functions linked to the innate and adaptive immunity.

Although the role of classical HLAs in HIV/HCV pathogenesis has been widely studied, the role of non-classical HLAs, such as HLA-G, HLA-F, and HFE, still remains enigmatic, despite their potential role in immune regulation and viral pathogenesis. It is critical to have a depth understanding of the role of these HLA alleles in viral infections (e.g., HIV and HCV). For instance, iron metabolism plays a complex but important role in immune regulation in the host. It appears that iron dysregulation, potentially through HFE, plays a larger role in the shift from oxidative phosphorylation to a glycolytic metabolism. It also seems to be highly involved in production of ROS leading to cell senescence with speculative effects on progression to AIDS and inflammaging. Therefore, any host genetic factors affecting iron regulation may impact viral pathogenesis.

Taken together, with the advancement in the available tools, larger GWAS and more in-depth analysis, SNP chip analysis of a larger number of SNPs across the region, and sequence-based genotyping of the HLAs would be essential to better delineate the role of HLA alleles in viral infections in terms of infection susceptibility or disease progression.

Finally, when designing vaccines or drugs from an epidemiological perspective, the major variants of certain genes such as HLA alleles and how these variants affect viral escape mutations, viral load heritability, and disease outcome should be considered.

## Author Contributions

NC wrote the first version of manuscript and designed Figures 1, 4 and 5. SE proposed the idea, designed the article, designed Figures 2 and 3, rewrote some sections, and revised and edited the whole manuscript.

## Conflict of Interest Statement

The authors declare that the research was conducted in the absence of any commercial or financial relationships that could be construed as a potential conflict of interest.
